# Predictors affecting vaccine hesitancy towards annual COVID-19 booster shots among populations from different countries

**DOI:** 10.1186/s12889-025-23047-x

**Published:** 2025-05-27

**Authors:** Sylvana Nady Gaber, Wafaa Y. Abdel Wahed, Bismark Jampim Abrokwah, Mohammed Ibrahim Al Hawamdeh, Lubna Abdelwahab Elsidigg, Ahmed A. Wegdan, Rasha H. Bassyouni

**Affiliations:** 1 Department of Medical Microbiology and Immunology, Faculty of Medicine, Fayoum University, Fayoum, Egypt; 2 Department of Public health & Community medicine, Faculty of Medicine, Fayoum University, Fayoum, Egypt; 3 Ghana Health Service/Ministry of Health, Accra, Ghana; 4 General director of Jordanian Experts for training, Amman, Jordan; 5 Directorate of Quality, Development and Accreditation, Federal Ministry of Health, Khartoum, Sudan

**Keywords:** Annual COVID-19, Booster shots, Vaccine hesitancy

## Abstract

**Background:**

Coronavirus Disease-19 (COVID-19) is reported to cause significant mortalities. Vaccination has the probability to reduce the burden of COVID-19. Annual vaccination is better to be established, but vaccine reluctance has been observed among different populations.

**Objectives:**

To recognize the associated factors and the predictors affecting vaccine hesitancy towards annual COVID-19 vaccine shots among African and Asian populations.

**Method:**

A cross-sectional study was conducted on a population from diverse nationalities using a structured, self-administered questionnaire. Adjusted odds ratios (ORs) and their 95% confidence intervals (CIs) were estimated using multiple logistic regression to identify predictors of vaccine hesitancy.

**Results:**

A total of 502 participants from four countries: 152 Jordanians (30.3%), 145 Egyptians (28.9%), 103 Ghanaians (20.5%), and 102 Sudanese (20.3%) were included in the study. The majority were females (307, 61.2%). Egyptians show the highest willingness to receive annual COVID-19 vaccine shots (99, 68.3%), while Jordanians (68, 44.7%), Ghanaians (42, 40.8%), and Sudanese (60, 58.8%) exhibit more hesitancy or refusal. Hesitancy is higher among the younger age group (56.5%, *P* = 0.003) and lower education levels (55.6%, *P* = 0.008). Higher knowledge (OR = 0.843, *p* = 0.002), and a positive attitude toward vaccine administration (OR = 0.878, *P* < 0.001) significantly predict lower hesitancy. The fear of severe side effects (42%) was the most common cause of COVID-19 vaccine hesitancy.

**Conclusions:**

Young age and low education levels are linked with increased hesitancy toward annual COVID-19 vaccination shots. Higher knowledge, and positive attitude, and previous influenza vaccination predict annual vaccine hesitance. Public health actions in the form of awareness campaigns are needed to promote the importance of COVID-19 booster shots vaccination and address worries about safety, and side effects to efficiently reach the target young and low education group with heighten vaccine service quality on the way to build vaccine assurance and lessen hesitancy.

**Supplementary Information:**

The online version contains supplementary material available at 10.1186/s12889-025-23047-x.

## Introduction

Severe acute respiratory syndrome coronavirus 2 (SARS-CoV-2) is a virus that causes coronavirus disease 2019 (COVID-19) [1]. The continuing COVID-19 pandemic has a considerable public health challenge with severe morbidity and mortality. In 2023–2024 more than 75,500 people died from COVID-19 [2]. SARS-CoV-2 has continuous genetic changes. Ongoing antigenic changes will pose a persistent risk to humans and increase the chance of recurrent epidemics or even pandemics [3]. With the absenteeism of actual therapy against COVID-19 and the decrease in vaccine protection over time, it is vital to get a yearly COVID-19 vaccine. Vaccine booster shots are essential in preventing the virus infection, lowering the chance of having a severe infection, decreasing the effects of long COVID-19, and protecting against miserable outcomes of COVID-19 [4]. Mass vaccination has long been considered the most effective line to fight infectious diseases [5]. Centers for Disease Control and Prevention (CDC) guidance, pointing out added shots of COVID-19 vaccines can improve fading immunity and defend against heart problems and post-Covid syndrome [6]. In a study done by Park et al. [7], they found that frequent COVID-19 booster vaccination (every 6–12 months) would efficiently diminish severe COVID-19 problems. As well, Bobrovitz et al. [8] suggested that added booster COVID-19 vaccine doses can recreate the level of protection despite the preceding decline.

Trust in vaccines is the main cause of the success of any vaccination project [9]. According to the World Health Organization (WHO) Strategic Advisory Group of Experts on Immunization Working Group delay in acceptance or refusal of vaccination despite availability of vaccination services” is well-defined as vaccine hesitancy [10, 11]. Vaccine refusal is determined by socio-cultural factors, individual knowledge, and risk perception. It is the task of scientists to understand the causes of vaccine hesitancy and to design better ways of communicating the benefits and hazards of COVID-19 vaccine booster shots [12]. Many studies have examined the willingness of high-income countries to take the COVID-19 vaccine, and some studies have involved middle-and low-income countries [9]. In 2021, a study done in 5 countries (Egypt, Palestine, Iraq, Saudi Arabia, and Sudan) found that 20.4% were hesitant to take the COVID-19 booster dose [13]. Another survey conducted on participants from Jordan, Kuwait, and Saudi Arabia revealed that 38.9% of participants would accept the COVID-19 vaccine, which may indicate a problem in fighting the COVID-19 pandemic [14].

Less is known, about yearly COVID-19 vaccine booster shots acceptance in middle- low-income countries. Understanding the factors of COVID-19 vaccine hesitancy is of universal concern because a delay in vaccination might result in the appearance and spread of novel variants [12]. The objective of the present study is to recognize the associated factors and the predictors affecting vaccine hesitancy towards annual COVID-19 vaccine shots among African and Asian populations to increase vaccine confidence.

## Methods

### Study design and participants

A cross-sectional study was conducted to explore factors that influence annual COVID-19 vaccine acceptance. The study population included adults aged 18 years and older from diverse nationalities, specifically Egyptians, Jordanians, Ghanaians, and Sudanese. Participants were recruited using an online survey distributed through numerous social media platforms and professional networks. The survey remained open for responses over three months, from May to August 2023.

### Sampling technique and size

A convenience sampling method was used to target individuals likely to provide diverse demographic and health-related data. Eligible participants were aged 18 years or older, residing in urban or rural areas, and willing to provide informed consent. Respondents were excluded if they failed to complete the survey or provided inconsistent responses.

The sample size for the study was calculated using Epi Info 7. Assuming a 50% prevalence of vaccine willingness, with a confidence interval of 95% and a precision of 5%, the minimum required sample size was 384. To account for potential non-responses, the sample size was increased by 20%.

### Data collection

Data were collected using a structured, self-administered questionnaire. The questionnaire was developed based on previous researches [10, 15, and 16]. The questionnaire was available in English for the Ghanaian population and in Arabic for the Egyptian, Jordanian, and Sudanese populations (Questionnaire version was uploaded as supplementary file). Initially, the questionnaire was developed in English and then translated into Arabic. To ensure clarity and accuracy, independent translators conducted both the translation and back-translation process.” It was pre-tested among 30 participants to ensure clarity and reliability. Based on their feedback, minor wording adjustments were made to improve clarity without altering the core content. Validity was assessed through expert review by Medical Microbiology, Immunology, and public health professionals. The Cronbach’s alpha values were computed: 0.71 for knowledge questions and 0.69 for attitude questions. The questionnaire involved four sections:

#### Demographics

Age, gender, marital status, residence, level of education, and occupation.

#### Health and COVID-19-related history

Presence of chronic diseases, pregnancy status (for females), prior COVID-19 infection, and whether participants had lost a family member due to COVID-19.

#### Vaccination behavior and perceptions

Compulsory vaccine, Influenza, and COVID-19 vaccine history, willingness to receive booster doses, and annual vaccine acceptance.

### Knowledge and attitudes

#### Knowledge

Participants answered 10 questions to assess their knowledge of COVID-19 vaccination. Each correct answer was awarded 1 point, while incorrect and “don’t know” responses were scored 0. The maximum possible score was 10. Questions addressed topics such as the vaccine’s ability to prevent disease, reduce severity and complications, build immunity, time-limited immunity, the necessity of booster doses, and contraindications (e.g., use in pregnancy or high fever). Additional items assessed vaccine efficacy against variants and whether prior infection negates the need for vaccination.

#### Attitudes

Attitudes and perceptions were evaluated using 15 statements. These included beliefs about vaccine effectiveness, immunity benefits, and safety concerns (e.g., allergic reactions and severe side effects). Participants also provided opinions on behavioral responses, such as adherence to precautionary measures post-vaccination, the importance of booster doses, vaccine efficacy against variants, and mandatory vaccination policies. Responses were scored as 0 for “disagree,” 1 for “neutral,” and 2 for “agree,” with a maximum possible score of 30.

An Exploratory Factor Analysis using Principal Component Analysis (PCA) with varimax rotation was done after data collection. The KMO measure (0.796) and Bartlett’s test (χ² = 3794.907, *p* < 0.001) confirmed that factor analysis was appropriate. Our analysis extracted four components explaining 45.68% of the total variance, supporting the distinction between knowledge and attitude constructs. The component matrix showed that knowledge-related item, and attitude-related items predominantly loaded onto separate factors, with minimal cross-loadings.

### Statistical analysis

Statistical analysis was conducted using SPSS version 23 (SPSS Inc., USA). Descriptive statistics were performed, with means and standard deviations (SD) calculated for quantitative data and frequencies and percentages reported for qualitative data. The proportion of participants willing to receive an annual COVID-19 vaccine in future years was estimated.

Knowledge and attitude scores were computed as follows: Knowledge: Correct answers scored 1, while incorrect and “don’t know” responses scored 0. The maximum score was 10. Attitudes: Responses were scored as 0 for “disagree,” 1 for “neutral,” and 2 for “agree,” with a maximum score of 30. Comparisons of knowledge and attitude scores across participant characteristics were performed using independent t-tests (for two groups) and ANOVA (for more than two groups). Inferential statistics, including chi-square (χ2) tests, were used to assess relationships between categorical variables and annual vaccine refusal. Adjusted odds ratios (ORs) and their 95% confidence intervals (CIs) were estimated using multiple logistic regression to identify predictors of vaccine hesitancy.

The dependent variable in this study was COVID-19 vaccine hesitancy, which was assessed based on participants’ responses to a question about their intention to receive annual COVID-19 vaccines. Participants who answered “No” or “Maybe” were classified as vaccine-hesitant, while those who responded “Yes” were categorized as vaccine-acceptant. The independent variables included demographic factors (gender, nationality, residence, education level, and employment in the medical sector), knowledge and attitude scores, history of influenza vaccination, history of COVID-19 vaccination, and whether a family member had died from COVID-19. Knowledge and attitude scores were treated as continuous variables, while the other independent variables were categorical.

Multicollinearity was assessed using Generalized Variance Inflation Factors (GVIFs), and model ft was evaluated using Nagelkerke pseudo-R2, (Nagelkerke R2 = 0.30), with GVIFs below 2, indicating no multicollinearity. Statistical significance was set at *P* < 0.05.

### Ethical considerations

#### Ethical approval

for the study was obtained from the Research Ethics Committee, Faculty of Medicine, Fayoum University No: R233. Participation was voluntary, and electronic informed consent was obtained before completing the survey. Participants were provided with detailed information about the study’s purpose, procedures, and data confidentiality before consenting. Confidentiality was maintained by anonymizing all responses. The study protocol followed the ethical principles and guidelines of The Declaration of Helsinki.

## Results

The results highlight key demographic and health-related information about respondents. Most are aged 31–50 years, predominantly female (61.2%), married (69.7%), and urban residents (77.7%). Education levels are high, with 62.9% holding postgraduate degrees, and 90.4% are employed. Nationalities include Jordanians (30.3%), Egyptians (28.9%), Ghanaians (20.5%), and Sudanese (20.3%). Health data shows 22.1% have chronic diseases, and 43.4% have children under 12. Among females, 4.2% are pregnant. About 30.5% of the participants work in the medical sector. Regarding COVID-19, 38.6% had been infected, 33.7% lost a relative due to SARS-CoV-2 virus infection, and 40.8% were not infected with the virus before (Table 1).

**Table 1:** Demographic and health characteristics of the participants

		*N*	%
Age in years	18–30	107	21.3
	31–40	142	28.3
	41–50	140	27.9
	51–60	58	11.6
	more than 60	55	11.0
Gender	female	307	61.2
	male	195	38.8
Marital Status	Married	350	69.7
	Single	124	24.7
	Widow/Divorced	28	4.6
Residence	Rural	112	22.3
	urban	390	77.7
Level of education	secondary education	9	1.8
	university education	177	35.3
	Postgraduate education	316	62.9
Occupation	Not Working	48	9.6
	Working	454	90.4
Nationality	Egyptian	145	28.9
	Jordan	152	30.3
	Ghana	103	20.5
	Sudan	102	20.3
Pregnancy status for females	pregnant	13	4.2
	Not pregnant	294	95.8
Have any chronic diseases	Yes	111	22.1
Having children less twelve years	Yes	218	43.4%
Do you work in the medical Sector?	Yes	153	30.5%
Did you previously get infected with COVID- 19 virus?	Maybe	103	20.5
	No	205	40.8
	Yes	194	38.6
Did you have any of your family members or relatives died from COVID-19?	No	333	66.3
	Yes	169	33.7

*N*: number; % percentage

Table 2 highlights the differences in vaccine perception and behavior across nationalities. Egyptians and Jordanians show higher acceptance of the influenza vaccine (41.4% and 48.0%, respectively) compared to Ghanaians (17.5%) and Sudanese (27.5%). Compulsory vaccine refusal is highest among Jordanians (19.7%) and Sudanese (16.7%), while Ghanaians (3.9%) and Egyptians (6.9%) are less likely to refuse. Most respondents completed their COVID-19 vaccination, with Jordanians leading (93.4%), followed by Ghanaians (88.3%), Egyptians (87.6%), and Sudanese (79.4%). The perceived risk of future COVID-19 infection is lowest among Ghanaians (54.4%) and Sudanese (52.0%), while Egyptians and Jordanians mostly perceive medium risk (48.3% and 53.9%). Egyptians show the highest willingness to receive booster doses (83.4%) and annual COVID-19 vaccines (68.3%) compared to Jordanians (44.7%) and Ghanaians (40.8%), and Sudanese (58.8%) as they exhibit more hesitancy or refusal. These findings highlight varying vaccine perceptions influenced by nationality.

**Table 2:** COVID-19 &influenza vaccination and risk perception status according to nationality

		Nationality								*P* value
		Egyptian		Jordan		Ghana		Sudan		
		*N*	%	*N*	%	*N*	%	*N*	%	
Recently, did you receive Influenza vaccine?	No	85	58.6%	79	52.0%	85	82.5%	74	72.5%	**0.000**
	Yes	60	41.4%	73	48.0%	18	17.5%	28	27.5%	
Did you ever refuse any compulsory vaccines for yourself or any family member?	No	135	93.1%	122	80.3%	99	96.1%	85	83.3%	**0.000**
	Yes	10	6.9%	30	19.7%	4	3.9%	17	16.7%	
Did you receive COVID 19 vaccines?	Yes. I receive only First dose	6	4.1%	8	5.3%	0	0.0%	8	7.8%	**0.001**
	Yes Complete course of the vaccine (2 shots scheduled vaccines or one-shot vaccine)	127	87.6%	142	93.4%	91	88.3%	81	79.4%	
	Not receiving the vaccine	12	8.3%	2	1.3%	12	11.7%	13	12.7%	
What do you think is the chance that you will get COVID-19 in the future?	No or low chance	42	29.0%	41	27.0%	56	54.4%	53	52.0%	**0.000**
	Medium chance	70	48.3%	82	53.9%	45	43.7%	36	35.3%	
	High chance	33	22.8%	29	19.1%	2	1.9%	13	12.7%	
Regarding the booster dose of the vaccine. If it is available, would you have the booster dose?	Maybe	7	4.8%	31	20.4%	20	19.4%	24	23.5%	**0.000**
	No	17	11.7%	36	23.7%	4	3.9%	8	7.8%	
	Yes	121	83.4%	85	55.9%	79	76.7%	70	68.6%	
If the COVID-19 vaccine become required yearly, do you agree to have it regularly?	may be	32	22.1%	47	30.9%	35	34.0%	15	14.7%	**0.000**
	No	14	9.7%	37	24.3%	26	25.2%	27	26.5%	
	Yes	99	68.3%	68	44.7%	42	40.8%	60	58.8%	

*N*: number; % percentage; Statistical significance: *P* < 0.05

Table 3 compares COVID-19 vaccine knowledge and attitude scores across the four nationalities. The mean knowledge score was highest among Egyptians (6.74 ± 2.2) out of 10, followed by Sudanese (6.5 ± 2.5), Ghanaians (6.04 ± 2.29), and Jordanians (5.9 ± 2.3), with a statistically significant difference (*P* = 0.014). In terms of attitude scores, Ghanaians scored the highest (22.5 ± 3.6), out of 30, followed by Sudanese (22.14 ± 4.6) and Egyptians (22.13 ± 3.8), while Jordanians had the lowest attitude score (20.43 ± 4.8).

**Table 3:** Comparison of COVID-19 vaccines knowledge and attitude scores among different nations

		Egyptian	Jordan	Ghana	Sudan	Total	*P* value
Knowledge score (10)	Mean ± SD	6.74 ± 2.2	5.9 ± 2.3	6.04 ± 2.29	6.5 ± 2.5	6.3 ± 2.3	**0.014**
Attitude score (30)	Mean ± SD	22.13 ± 3.8	20.43 ± 4.8	22.5 ± 3.6	22.14 ± 4.6	21.7 ± 4.3	**0.000**

Statistical significance: *P* < 0.05

Table 4 identifies factors influencing hesitancy toward annual COVID-19 vaccination shots. Hesitancy is higher among younger age groups (56.5% for ages 18–30) and decreases with age (*P* = 0.003). Ghanaians (59.2%) and Jordanians (55.3%) exhibit higher hesitancy compared to Sudanese (41.2%) and Egyptians (31.7%) (*P* < 0.001). Postgraduate education reduces hesitancy (41.1%) compared to lower education levels (about 55%), *P* = 0.008. Married individuals are less hesitant (41.7%) than divorced (65.0%) and single participants (*P* = 0.007). Those perceiving a high chance of infection (36.4%) or experiencing a relative’s death from COVID-19 (32.5%) are less hesitant, as are recent influenza vaccine recipients (33.0%) (*P* < 0.001). Higher knowledge (7.03 ± 2.003) and attitude scores (22.99 ± 2.99) relate with lower hesitancy (*P* < 0.001). Gender, residence, medical sector work, and chronic diseases showed no significant impact.

**Table 4:** Factors affecting annual COVID-19 vaccine refusal and hesitancy

		Annual vaccine				*P* value
		Refusal and hesitancy		Acceptance		
		*N*	%	*N*	%	
Age in years	18–30	61	56.5%	47	43.5%	**0.003**
	31–40	73	51.4%	69	48.6%	
	41–50	56	40.3%	83	59.7%	
	51–60	28	48.3%	30	51.7%	
	more than 60	15	27.3%	40	72.7%	
Nationality	Egyptian	46	31.7%	99	68.3%	**0.000**
	Jordan	84	55.3%	68	44.7%	
	Ghana	61	59.2%	42	40.8%	
	Sudan	42	41.2%	60	58.8%	
Education level	Secondary education	5	55.6%	4	44.4%	**0.008**
	University education	98	55.4%	79	44.6%	
	Post graduate	130	41.1%	186	58.9%	
Gender	Female	144	46.8%	164	53.2%	0.85
	Male	89	45.9%	105	54.1%	
Marital Status	Divorced	13	65.0%	7	35.0%	**0.007**
	Married	146	41.7%	204	58.3%	
	Single	71	57.3%	53	42.7%	
	Widow/Widower	3	37.5%	5	62.5%	
Residence	Rural	56	50.0%	56	50.0%	0.388
	Urban	177	45.4%	213	54.6%	
Working in medical sector	No	165	47.1%	185	52.9%	0.62
	Yes	68	44.7%	84	55.3%	
Having children less twelve years	No	125	43.9%	160	56.1%	0.188
	Yes	108	49.8%	109	50.2%	
Have any chronic diseases	No	188	48.1%	203	51.9%	0.16
	Yes	45	40.5%	66	59.5%	
What do you think is the chance that you will get COVID-19 in the future?	No or low chance	83	43.0%	110	57.0%	**0.023**
	Medium chance	122	52.6%	110	47.4%	
	High chance	28	36.4%	49	63.6%	
Did you have any of your family members or relatives died from COVID-19?	No	177	53.5%	154	46.5%	**0.000**
	Yes	55	32.5%	114	67.5%	
Recently, did you receive Influenza vaccine?	No	174	53.9%	149	46.1%	**0.000**
	Yes	59	33.0%	120	67.0%	
Knowledge score	Mean ± SD	5.5	2.4	7.03	2.003	**0.000**
Attitudes score	Mean ± SD	20.18	5.08	22.99	2.99	0.000

*N*: number; % percentage; Statistical significance: *P* < 0.05

Table 5 identifies predictors linked with COVID-19 vaccination hesitancy. Compared to Egyptians (reference group as it is categorized as African and Asian countries), participants from Jordan (OR = 2.137 (1.19–3.83), *p* = 0.011) and Ghana (OR = 2.140(1.156–3.96), *p* = 0.015) were significantly more likely to exhibit vaccine hesitancy. Sudanese participants showed no significant difference (*p* = 0.478). Higher knowledge scores (OR = 0.843 (0.76–0.94), *p* = 0.002) and positive attitudes (OR = 0.878(0.83–0.93), *p* < 0.001) significantly reduced vaccine hesitancy. Those who had not received an influenza vaccine were more likely to be hesitant (OR = 0.482 (0.31-0.75), *p* = 0.001). Those who had not received an influenza vaccine were more likely to be hesitant (OR = 0.42 (0.27–0.67). Gender, residence (rural vs. urban), working in the medical sector, education level, and receiving the COVID-19 vaccine showed no significant predictor of vaccine hesitancy.

**Table 5:** Predictors associated with COVID-19 vaccination hesitance among study participants (Multivariate logistic regression analysis) Nagelkerke R Square 0.307

Predictors		B	*P* value	AOR	95% C.I.for OR	
					Lower	Upper
Gender	Male			1		
	Female	0.047	0.829	1.048	0.683	1.608
Nationality	Egyptian (reference group)		**0.022**	**1**		
	Jordan	0.759	**0.011**	2.137	1.193	3.826
	Ghana	0.761	**0.015**	2.140	1.157	3.957
	Sudan	0.216	0.478	1.241	0.683	2.255
Residence	Urban			1		
	Rural	-0.205	0.436	0.815	0.486	1.365
Working in medical sector	No			1		
	yes	-0.166	0.472	0.847	0.539	1.332
Education level	Post graduate education			1		
	(lower educational level) Vs post graduate)	-0.177	0.417	0.838	0.546	1.285
Knowledge score		-0.171	0.002	0.843	0.756	0.939
Attitudes score		-0.130	< 0.001	0.878	0.826	0.933
Receiving influenza vaccine	Yes			1		
	No	-0.730	0.001	0.482	0.309	0.752
Have any of your family members or relatives died from COVID-19?	Yes					
	No	-0.865	< 0.001	0.421	0.266	0.667
Receiving COVID-19 vaccine (no Vs, yes)	Yes			1		
	No	-0.349	0.375	0.705	0.326	1.526
	Constant	4.787	0.000	119.934		

Statistical significance: *P* < 0.05

**Fig. 1:**
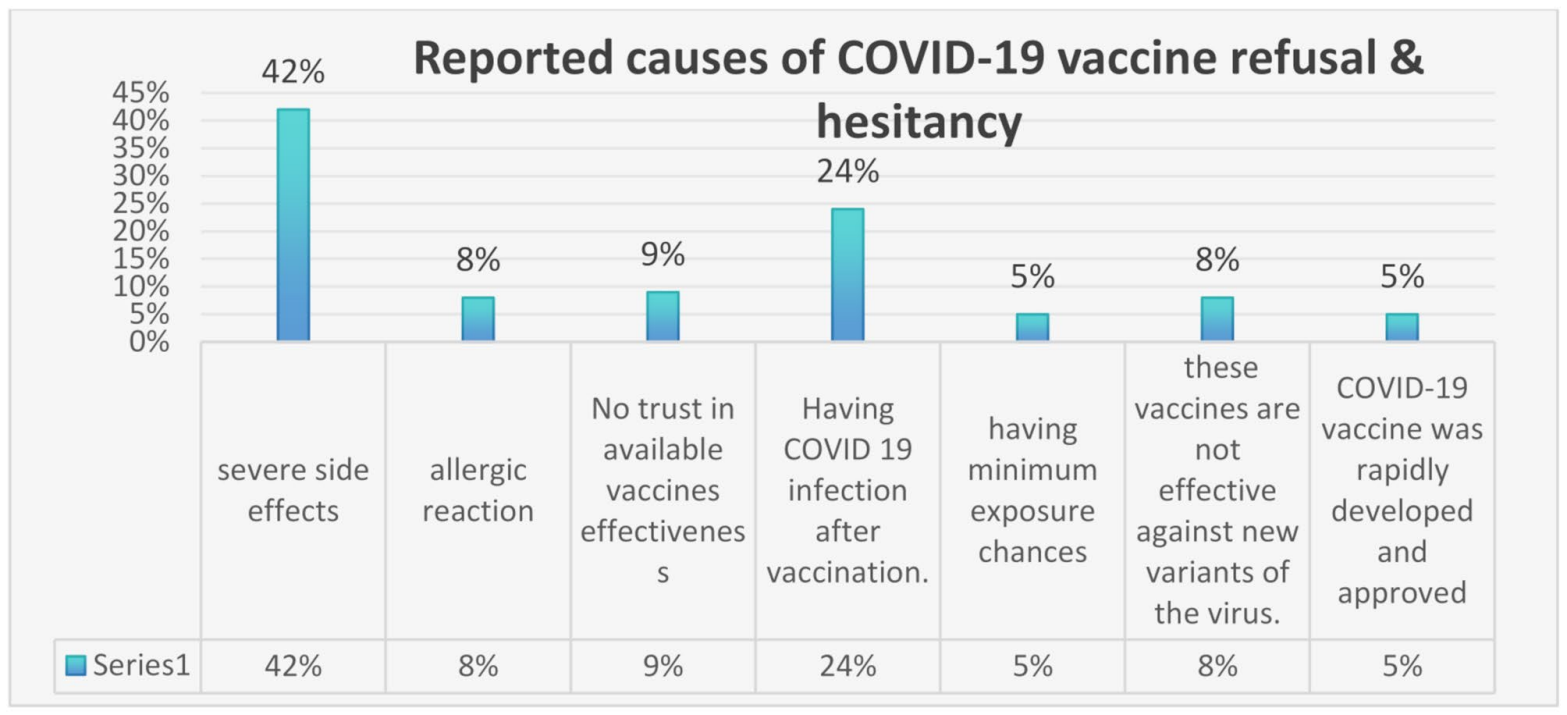
Reported causes of annual COVID-19 vaccination refusal

Our participants reported some factors that caused COVID-19 vaccine hesitancy; the most detected cause was concerns about severe side effects (42%), followed by contracting COVID-19 after vaccination (24%) (Fig. 1).

## Discussion

The appearance of SARS-CoV-2 in late 2019 generated the global (COVID-19) pandemic in its fifth year. Vaccines were established and have saved millions of lives. However, the occurrence of periodic SARS-CoV-2 antigenic changes may result in a staggering number of morbidity and mortality among humans [17]. Reformulate Vaccine booster shots is still a vital way to decrease disease-specific severity and mortality rates [18]. Despite of the confirmed efficiency of vaccination, many individuals who remain unvaccinated [19]. The vaccination process has been met with an undesirable, phenomenon known as vaccine hesitancy [20].

We conducted a cross-sectional study to recognize factors affecting the acceptance or refusal of the annual COVID-19 vaccination shots among the populations from Egyptians (28.9%), Jordanians (30.3%), Ghanaians (20.5%), and Sudanese (20.3%), if annual vaccination is recommended by national or international bodies.

Regarding the rate of COVID-19 vaccination, 93.3% of the Jordanians completed the full doses of the vaccine schedule followed by Ghanaians (88.3%), Egyptians (87.6%), and Sudanese (79.4%). In partial agreement with the present results, Al-Qerem and Jarab [21] found that 70% of Jordanian participants received the full doses of COVID-19 vaccine while Kandeel et al. [22] demonstrated that 44.3% of Egyptians were fully vaccinated. A survey performed by Lazarus et al. [23] reported that the rate of COVID-19 vaccination across 23 countries was 79.1%. Lower rates were reported by other studies; Masoud et al. [15] revealed that Jordan fall in the second rank with vaccination rate (49.5%) followed by Egypt (45.5%). Lataifeh et al. [16] reported COVID-19 vaccination rates range from 20. % to 94. % in European nations (Germany, France, Poland, and Italy) while Middle East and African countries demonstrated lower rates below 50%. Al Rahbeni et al. [24] reported that among Northern Africa population prevalence of COVID-19 vaccine acceptance was (24.28%).

In the present study, Egyptians are most likely to accept both COVID-19 booster doses (83.4%) and yearly vaccination (68.3%). Furthermore, we found that Sudanase has the highest hesitancy to yearly vaccination (26.5%), and Jordanians show reluctance, with 23.3% rejecting the booster and 24.7% refusing yearly vaccination despite their high acceptance of full COVID-19 doses in the primary vaccination schedule, this is may be due to their belief in the ineffectiveness of the vaccines towards the new variants. This is nearly in consistent with a survey done across 53 countries to determine the level of vaccine hesitancy which found that average levels of hesitancy were 47.4% among the Middle Eastern and North African population then 26.2% among East Asia and Pacific people and 15.5% among sub-Saharan Africa countries [25]. Diverse studies reported survey results from low and middle-income countries [9, 26, 27]. Also Kanyanda et al. [28] found that vaccine acceptance in 6 sub-Saharan African countries varieties from 97.9% in Ethiopia to below to 64.5% in Mali.

Influenza vaccination is reported to be effective in preventing influenza infection and decreasing disease severity, also there are emerging data suggesting that immunization against influenza is also protective against severe COVID-19, which concludes that protection against seasonal influenza and COVID-19 infection could be intertwined [29]. By comparing the vaccination rates among our participants, the present finding showed that less than half of Egyptians (58.6%) and Jordanians (52%) received the annual influenza vaccine compared to Ghanaians (82.5%) and Sudanese (72.5%). These results were comparable with previous studies reported the vaccination rate in Egypt was 46.8% [30], and 52.9% in Jordan [31]. Also, Sansone et al. [32] found that two-thirds of the responders in Italy had been vaccinated against seasonal influenza.

The interesting results in the present study are the discrepancies in influenza and COVID-19 vaccination rates among Egyptians (58.6% and 87.6% respectively) and Jordanians (52% and 93.3% respectively), while they are comparable among Ghanaians and Sudanese. Similarly, Domnich et al. [33] reported that 85.1% of the study’s participants in Italy had completed the primary schedule of COVID-19 vaccination, while only 42.6% had received the seasonal influenza vaccine. These results revealed that changing vaccine perceptions are influenced by nationality.

Our findings showed that Ghanaians (59.2%) and Jordanians (55.3%) exhibit higher hesitancy to the annual COVID-19 vaccine. The variation in COVID-19 vaccine hesitancy across countries could reflect the lack of trust in the healthcare system and the effect of the governments. Consequently, needed more research to understand differences in vaccine hesitancy across regions.

There is an increasing need to identify and understand factors affecting the acceptance rate and the obstacles that interfere with vaccine uptake. So, we investigated several factors influencing annual vaccine acceptance. We found that hesitancy is higher among the younger age groups (56.5% for ages 18–30) and decreases with age (*P* = 0.003). This is consistent with Masoud et al. [15] in a study conducted on populations from six countries (Egypt, Tunisia, Palestine, Sudan, Jordan, and Yemen) who found that individuals above the age of forty were more likely to accept vaccination. Alatrany et al. [34] in Iraq reported increased vaccination rates with age. Also, Lazarus et al. [23] stated that booster hesitancy among vaccinated populations was associated with younger age in Germany, France, South Korea, Poland, Sweden, and Spain. Truly, young generations have a perspective view they have a higher level of protection against viruses compared to the old one [35]. Also, Severe illness, and those with underlying comorbidities in the elderly, are at high risk for COVID-19 infection mortality [29], this may explain this finding.

Hesitancy was also decreased with low education level (55.6%), Li et al. [36] revealed that people with higher educational levels have higher intentions of accepting vaccines. Husseina et al. [37] found that 54% of the participants had a university degree. Sansone et al. [32] found that 41.2% of their responders had a post-graduate degree. Also, in terms of education level, Zhao et al. [38] demonstrated higher educational achievement was associated with influenza vaccine acceptance.

Brackstone et al. [39] demonstrated that among Malaysian participants vaccine confidence was associated with higher education (OR, 1.30, 95% CI, 1.03–1.66; *p* < 0.028). A review on determinants of COVID-19 vaccines in low and middle-income countries also reported that higher education had lower hesitancy of COVID-19 vaccines [40]. A better understanding of the disease’s hazard and the vaccine’s profits by these populations possibly reflect this finding [24].

The results that younger adults and those with low education levels are more vaccine hesitant are consistent with findings of other studies presented by [26, 41].

Married individuals are less hesitant than singles, this could be due to the vaccination booster shots giving them a sense of security from catching the disease for them and their children. In partial agreement with the present results, Alatrany et al. [34] found that COVID-19 vaccination rates increased with married, divorced, or widowed persons, and those having children.

Although, people from different regions may have different recognition and attitudes toward the diseases and vaccines, accounting for the degrees of vaccine acceptance between regions [29], residence, in the current study showed no significant impact on vaccine acceptance.

Omar and Amer [42] showed that the knowledge score among Egyptians was increased and the majority of the participants had a positive attitude toward vaccination acceptance. Our participants have enlarged knowledge scores. In the current study higher knowledge was one of the factors associated with the acceptance rates. For attitude scores, Egypt, Ghana, and Sudan have similar average increased scores. In line with our finding, Darbandi et al. [43] revealed that the principal reasons for accepting the COIVD-19 vaccine were a heightened perception of risk associated with the virus and a general trust in the healthcare system. Hakim et al. [30] also found that there was a significant positive association between attitude score and vaccine uptake.

In Malaysia, Kyaw et al. [35] stated that there are numerous perspectives regarding the vaccine and many associations got to be linked with the hesitancy to the vaccination whether it can be from social media influence, knowledge level and reading ability of literature.

Many factors can influence the level of knowledge, and attitude and contribute to vaccine hesitancy, including socioeconomic status, and perceived hazard. Socioeconomic status is determined by factors such as income, occupation, and education. Philip [44] proposed that individuals with lower socioeconomic status are more likely to be vaccine-hesitant. This may be due to limited access to healthcare services, or a lack of information about vaccines. Also, people who perceive the threat of COVID-19 as low may be more hesitant to get vaccinated. This may be due to misinformation or a lack of understanding of the severity of the disease.

Stamatatos et al. [45] found that the most commonly reported general side effects for COVID-19 vaccines were fatigue, body aches, fever, and headache. Masoud et al. [15] and Kandeel et al. [22] established that vaccine hesitancy was due to fear of adverse events (17.5%), this is in consistency with our findings that the most common cause of vaccine refusal is the fear of side effects (15.6%). Similarly, Pires [46] demonstrated that strong beliefs that the COVID-19 vaccines would cause side effects or be unsafe are strong predictors of vaccine hesitancy.

Fox et al. [47] found that the hazard of COVID-19 vaccines was a significant predictor of vaccine refusal. Also, Darbandi et al. [43] established the most frequently cited reasons for vaccine hesitancy is the potential dangers of the vaccines, and the possibility of adverse effects (such as infertility or death). Hakim et al. [30] found that the most identified reason for non-compliance was a lack of trust about vaccine efficacy and its effects.

To identify factors that predict annual COVID-19 vaccination acceptance or hesitancy, we performed a multiple forward stepwise logistic regression analysis. By nationality, we found Jordanians and Ghanaians were 2.1 more likely to exhibit vaccine hesitancy than Egyptians (*p* > 0.05). Higher knowledge scores and positive attitudes significantly reduced vaccine hesitancy by more than 0.8 times (*p* > 0.05). Also, those who never received an influenza vaccine before were more likely to be hesitant (OR = 0.482, *p* = 0.001). Previous data also suggests that previous seasonal influenza vaccination is a strong predictor of COVID-19 vaccination acceptance [46, 48–50]. In the same line with the present results, Kebede and Aytenew [51] reported that, good knowledge, positive attitude, and higher educational level were strong predictors of primary COVID-19 vaccination acceptance.

Lastly, Future research like longitudinal studies should focus on assess long-term vaccine acceptance and the impact of public health involvements on hesitancy and to understand differences in vaccine hesitancy across regions.

### Limitations of the study

The use of convenience sampling in this study introduces selection bias, which may result in an unrepresentative sample across the four countries studied. This limitation affects the generalizability of the findings. Furthermore, the reliance on an online questionnaire may have excluded individuals with limited internet access, further impacting the study’s representatives. Also, the questionnaire with closed-ended answers has lost some relevant concerns. It lacks questions on vaccine information they get and the trust of these sources. Moreover, the limited range of the low number of our participants from four countries: Egypt, Jordan, Sudan, and Ghana may not be represented by all African and Middle-East populations. Coming studies might use a larger number of responders and different measures of vaccine hesitancy.

## Conclusions

Egyptians have the highest acceptance rate for the yearly COVID-19 vaccine. Younger adults and those with low education levels are more vaccine-hesitant. Higher knowledge, positive attitude, and nationality were considered the main predictors for annual vaccine acceptance. It is important to start relevant public health measures through the motivation of awareness campaigns to promote the importance of booster shots vaccination and to address worries about safety, and side effects to improve the yearly vaccine acceptance if recommended by national or international bodies. Continuous research to understand vaccine perceptions and to report the needs of varied populations among different countries is needed. Increasing the population’s trust in the government and vaccine manufacturers are vital concerns in aiding in the formulation of active vaccination policies.

## Electronic supplementary material

Below is the link to the electronic supplementary material.

Supplementary Material 1

## Data Availability

All data generated or analyzed during this study are included in this article.

## Abbreviations

COVID-19: Coronavirus Disease-19
SARS-CoV-2: Severe acute respiratory syndrome coronavirus 2
SD: Standard deviations
ORs: Odds ratios
CIs: Confidence intervals
CDC: Centers for Disease Control and Prevention
N: Number of isolates
%: Percentage
SPSS: Statistical package for social science
