# Supplementary material for: Predictors affecting vaccine hesitancy towards annual COVID-19 booster shots among populations from different countries
Source: BMC Public Health. 2025 May 27;25:1953. doi: 10.1186/s12889-025-23047-x (PMC12107994; doi:10.1186/s12889-025-23047-x)
Supplement: Supplementary file 1 — Supplementary Material 1 [file 12889_2025_23047_MOESM1_ESM.doc]

窗体顶端

Acceptance of Annual booster shots) for Covid19 vaccine questionnaire

**Nationality***

**. Age in years**

18-30

31-40

41-50

51-60

more than 60

**.Gender***

male

female

**Marital Status***

Single

Married

Divorced

Widow/Widower

**Residence***

Rural

Urban

**Level of education***

primary education

preparatory education

secondary education

university education

post graduate

**Occupation***

no work

student

governomental employee

work in private sector

farmer/worker

skilled worker

unregular work

others

**.Have any chronic diseases***

yes

no

if yes , specify

diabetes

hypertension

lung disease

heart diseases

cancer

liver diseases

kidney diseases

autoimmune disease

others

**Pregnancy Status (for females)**

pregnant

not pregnant

**Having children less twelve years***

yes

no

**Do you work in the medical Sector?***

yes

no

**Did you previously  get infected with Covid 19 virus?***

Yes

No

Maybeا

**Did you have any of your family members or relatives died from COVID-19?***

Yes

No

**Recently, did you receive Influenza vaccine?***

Yes

No

**Did you ever refuse any compulsory vaccines for yourself or any family member?***

Yes

No

**Did you receive COVID 19 vaccines?***

1. Yes . I receive only First dose

2. Yes Complete course of the vaccine (2 shots scheduled vaccines or one-shot vaccine)

3. Not receiving the vaccine

**Did you suffer any side effects after vaccination?**

Yes

No

**What did you suffer?**

fever

headache

fatigue

Dizziness

Local reaction at site of injection

Thrombus formation

Respiratory distress

Flu-like symptoms

others

fever

headache

fatigue

Dizziness

Local reaction at site of injection

Thrombus formation

Respiratory distress

Flu-like symptoms

others

**What do you think is the chance that you will get COVID-19 in the future?***

a. No or low chance

b. Medium chance

c. High chance

**Do you have any information about Covid 19  vaccines?***

Yes

No

Source of information about COVID vaccines

social media

mass media

friends and relatives

training couses

health care workers

others

social media

mass media

friends and relatives

training couses

health care workers

others

**窗体顶端**

Please check  the best answer that describe your knowledge

|  | yes | no | | don’t know |  |
| --- | --- | --- | --- | --- | --- |
| COVID vaccines can prevent disease |  |  | |  |  |
| COVID vaccines Decrease severity or complication |  |  | |  |  |
| the vaccine help to build your immunity |  |  | |  |  |
| Immunity after vaccination is time limited. |  |  | |  |  |
| There is no need to get a booster dose of COVID-19 vaccine |  |  | |  |  |
| COVID vaccines are contraindicated in pregnant |  |  | |  |  |
| COVID vaccines are contraindicated in chronic disease patients |  |  | |  |  |
| Highly feverish people shouldn't receive vaccine |  |  | |  |  |
| previously infected COVID-19 shouldn’t receive the vaccine |  |  | |  |  |
| COVID-19 vaccines isn't effective against the new variants of the virus |  |  |  | | |

Attitudes and perception part, please choose the answer that describe your attitude or perception

|  | Agree | neutral | disagree |
| --- | --- | --- | --- |
| The vaccine is effective in preventing the disease. |  |  |  |
| The vaccine is effective in reducing risk of developing serious illness and death, |  |  |  |
| vaccines would produce immunity against COVID infection |  |  |  |
| Everyone should receive the vaccine. |  |  |  |
| I think getting immunity after infection is better than vaccination. |  |  |  |
| COVID-19 vaccines may cause allergic reaction |  |  |  |
| COVID-19 vaccines may cause severe side effects |  |  |  |
| receiving the vaccine will not make me reduce precautionary measures and lead my life normally |  |  |  |
| the vaccinations available in my country are not effective or safe |  |  |  |
| no need the vaccine if I got the infection. |  |  |  |
| It is possible to catch Covid 19 infection even after vaccination. |  |  |  |
| booster dose of COVID-19 vaccine is important to to keep me immune |  |  |  |
| COVID vaccines are no effective against new variants of the virus |  |  |  |
| you will recommend their family members to take COVID-19 vaccines |  |  |  |
| COVID vaccines should be mandatory to all people |  |  |  |

Regarding the booster dose of the vaccine. If it is available, would you have the booster dose?*

Yes

No

Maybe

if  you do not agree to receive the booster dose

- I got Covid 19 infection after 2 shots of vaccination.

- I am afraid from severe side effects

- I am afraid from allergic reaction

- I don’t trust in the effectiveness of the vaccine

- I don’t trust in available vaccines types in my country

- I think these vaccines are not effective against new variants of the virus.

- I have minimum exposure chances

COVID-19 vaccine was rapidly developed and approved

others

If the Covid 19 vaccine become required yearly, do you agree to have it regularly?*

Yes

No

may be

Back
